# A map of brain neuropils and fiber systems in the ant *Cardiocondyla obscurior*

**DOI:** 10.3389/fnana.2014.00166

**Published:** 2015-02-04

**Authors:** Joris M. A. Bressan, Martin Benz, Jan Oettler, Jürgen Heinze, Volker Hartenstein, Simon G. Sprecher

**Affiliations:** ^1^Department of Biology, Institute of Developmental and Cell Biology, University of FribourgFribourg, Switzerland; ^2^Biologie I, Universität RegensburgRegensburg, Germany; ^3^Department of Molecular, Cell and Developmental Biology, University of CaliforniaLos Angeles, CA, USA

**Keywords:** neuropile compartements, *Cardiocondyla obscurior*, neuroanatomy, fiber tracts, hymenoptera

## Abstract

A wide spectrum of occupied ecological niches and spectacular morphological adaptations make social insects a prime object for comparative neuroanatomical studies. Eusocial insects have evolved complex societies based on caste polyphenism. A diverse behavioral repertoire of morphologically distinct castes of the same species requires a high degree of plasticity in the central nervous system. We have analyzed the central brain neuropils and fiber tract systems of the worker of the ant *Cardiocondyla* *obscurior*, a model for the study of social traits. Our analysis is based on whole mount preparations of adult brains labeled with an antibody against *Drosophila*-Synapsin, which cross-reacts strongly with synapses in *Cardiocondyla*. Neuropil compartments stand out as domains with a certain texture and intensity of the anti-Synapsin signal. By contrast, fiber tracts, which are composed of bundles of axons accompanied by glia and are devoid of synapses, appear as channels or sheaths with low anti-Synapsin signal. We have generated a digital 3D atlas of the *Cardiocondyla* brain neuropil. The atlas provides a reference for future studies of brain polymorphisms in distinct castes, brain development or localization of neurotransmitter systems.

## Introduction

Social Hymenoptera (ants and many wasps and bees) are characterized by their social life style and the formation of colonies. Their ecological success is based on a highly elaborate division of labor between different female phenotypes or castes. In general these different phenotypes are specialized for reproduction or for non-reproductive helping. Both behaviors are under the control of the central nervous system.

The high degree of behavioral plasticity within the same species in turn must be based on differences and modifications in brain architecture. The analysis of caste-specific brain structure and development in social insects promises to provide insight into the mechanism by which the expression of genes is linked to a specific neural phenotype. A wealth of previous works has already documented intraspecific brain polymorphism between different castes of social insects (Zube and Rössler, 2008; Molina et al., 2009; Mysore et al., 2009; Kuebler et al., 2010; Nakanishi et al., 2010; Smith et al., 2010). The size of certain brain compartments, notably the antennal lobe (AL), optic lobe, mushroom body (MB) and central complex (CCX), varies among different worker castes and is generally correlated with task plasticity (Withers et al., 1995; Farris et al., 1999, 2001; Farris and Strausfeld, 2001; Marin et al., 2002; Farris and Sinakevitch, 2003; Ismail et al., 2006; Jefferis et al., 2007; Cachero and Jefferis, 2008; Muscedere and Traniello, 2012). It has been proposed that the complexity of behavioral tasks associated with sociality has led to the enlargement of compartments involved in associative learning, in particular the MB. This trend has been observed both across as within species when comparing brains of different behavioral castes (Fahrbach, 2006; Smith et al., 2010). Differences in sizes of compartments, reflecting neuron numbers and neurite complexity, could be due to developmental mechanisms or to experience. In many cases, neuroanatomical polymorphisms between castes become apparent during metamorphosis; prominent examples are the caste-specific patterns of antennal glomeruli (Withers et al., 1995; Seid and Wehner, 2009; Kuebler et al., 2010). Likewise, the relative scarcity of synapses and smaller glomerular volumes in queen bees compared to worker bees is brought about by a developmental heterochrony (earlier differentiation of glomeruli in queens; Groh and Rössler, 2008). That direct experience plays a role in at least some instances was shown in studies of workers in the desert ant *Cataglyphis fortis*. Here, visual experience, rather than hard-wired developmental mechanisms, was responsible for pruning and dendrite extension in the MB of individuals that switched to take over tasks outside the nest (Seid and Wehner, 2009; Stieb et al., 2010).

Comparative descriptions of insect brains mainly focus on a few well known compartments of the brain, and mostly do not address neuroanatomical features of other neuropils, simply because they are difficult to recognize. Exceptions to this are the advances that have been made in characterizing the brain of the honeybee, *Apis mellifera*. The honeybee has been studied for many decades in view of its intriguing behaviors, such as its surprising capacities in navigation, intra-species communication, visual and olfactory learning and memory formation (Ribbands et al., 1952; Menzel, 1983; Menzel and Muller, 1996; Menzel et al., 1996; Menzel and Giurfa, 1999, 2001; Srinivasan, 2010; Menzel and Greggers, 2013). This large body of knowledge on honeybee behavior has been complemented by the identification of many behaviorally relevant neurons. Integrating this information in a standardized 3D atlas provides an important tool to understand brain circuits (Brandt et al., 2005). This approach has further been pursued by Menzel, Rybak and colleagues who developed the “The honeybee standard brain” (HSB) database, an interactive tool to integrate morphologies and the reference atlas of neurons in the honeybee brain (Brandt et al., 2005; Rybak et al., 2010). The approach to develop a 3D standard brain has also been used for other insects, e.g., the fruit fly, *Drosophila melanogaster* (Rein et al., 2002; Ito et al., 2014), the hawkmoth, *Manduca sexta* (El Jundi et al., 2009b), the desert locust, *Schistocerca gregaria* (Kurylas et al., 2008; El Jundi et al., 2009a), the red flour beetle, *Tribolium castaneum* (Dreyer et al., 2010) and the monarch butterfly *Danaus plexippus* (Heinze and Reppert, 2012; Heinze et al., 2013). For most other insects, however, the few above-mentioned compartments are recognizable by their characteristic modular internal architecture and provide often the only anatomical reference points. Extending our knowledge to include more anatomical features, including tracts and compartments, will be helpful to analyze brain function.

In the present paper we have analyzed the pattern of tracts in relation to brain compartments in the ant *Cardiocondyla obscurior*. This ant is particularly interesting in view of phenotypic plasticity because of the unusual co-occurrence of the standard queen-worker polyphenism and a polyphenism of winged and wingless males (Schrempf and Heinze, 2006; Oettler et al., 2010). Whole mount preparations of adult brains were labeled with anti-*Drosophila*-Synapsin, an antibody that visualizes synapses in the *Drosophila* brain, and that cross-reacts strongly with synapses in *Cardiocondyla*. Neuropil compartments stand out as domains with high levels of anti-Synapsin signal. Compartment boundaries and individual tracts, composed of bundles of axons accompanied by glia and devoid of synapses, appear as channels or sheaths with low anti-Synapsin signal. We generated a digital 3D model of the tracts of the *Cardiocondyla* brain. In this paper we specifically focus on the adult *Cardiocondyla* worker caste, providing a brain map that, in future studies, will be extended to other castes, and multiple developmental stages.

## Materials and methods

### Animals

*Cardiocondyla* colonies were collected from decaying coconuts in an experimental plantation in Una, Bahia, Brazil in July 2009. They were housed in small petri dishes with a plaster floor and small indentations in the plaster that were covered with a microscope slide and dark red plastic serving as nest sites. The colonies were kept in the laboratory in an incubator at 30°C and 12 h/12 h light cycles. The colonies were fed twice a week with honey and crickets (from the local supermarket and pet store). Worker animals for dissection were taken from the nests of colonies.

### Antibody specificity information

For anatomical analyses we used an antibody against *Drosophila* Synapsin (SYNORF1) (Klagges et al., 1996; Table 1). Synapsins are phosphoproteins, which are associated with synaptic vesicles and are involved in controlling neurotransmitter release. SYNORF1, anti-Synapsin, detects an epitope that is widely conserved in arthropods and labels not only presynapses in insects but also in crustaceans and spiders (Fabian-Fine et al., 1999; Harzsch et al., 1999). This antibody is available at the Developmental Studies Hybridoma Bank (DHSB). We further used an antibody against acetylated Tubulin (Sigma, product Number: T7451; Batch Number: 062M4841V) to positively label nerve bundles and fibers.

**Table 1:** **Comparative summary of the axon fascicles associated with compartments boundaries in *Drosophila* and in *Cardiocondyla obscurior***.

Name	Axon fascicle			Compartment boundboundaries	
		***Droso*.**	***Cardio*.**	***Drosophila***	***Cardiocondyla***
Antennal lobe tract	ALT	√	√	CCX-IPm/v
medial	mALT	√	√		ML-VL
lateral	IALT	√	√
Medial equatorial fascicle	MEF	√	√	CCX-IPm/v	IP-VMC
Longitudinal superior-medial fascicle	loSM	√	√	SMP-IPm
Posterior	loSMp	√	√		SMPp-IPp
Anterior	loSMa	√	√		SMPa-IPa
Lateral equatorial fascicle	LEF	√	√
Posterior division	LEFp	√	-	IP-VLP-VMC
Anterior division	LEFa	√	√	IP-VLP-LAL	IP-VLP-LAL
Posterior lateral fascicle	PLF	√	√	loILp
Longitudinal ventral fascicle	loV	√	√
Posterior division	loVP	√	√	PLP-VMC
Anteromedial subdivision	loVM	√	√	VMCpr-PONP	VMCpr-SOG
Anterointermediate division	loVI	√	-	AL/VMCpr-AMMC
Anterolateral division	loVL	√	√	LAL-VLPa	AMMC-VMC
Transverse superior-anterior fascicle	trSA	√	√	SLPa-IPI	SLP-IP
Transverse superior-intermediate fascicle	trSI	√	√	SLPp-IPI
ventral division	trSlv	-	√		SLP-IP(I)
dorsal division	trSId	-	√		SMP-IP
Transverse superior-posterior fascicle	trSP	√	√		SMPp-IPp
medial division	trSPm	√	-	SLPp-SMP
				SMPp-IPm
lateral division	trSPI	√	-	SLPp-LH
				SLPp-IPI
Great commissure	GC	√	√	VMCpr-po-in-su	VMCpr-po-in-su
Lateral accessory lobe commissure	LALC	√	√	LALdo-ven	LALdo-ven

### Immunohistochemistry and antibody staining procedure of adult brains

For immunostainings heads of adult *Cardiocondyla* workers were dissected on a silicon plate in a drop of cold 1xPBS. PBS is kept on ice.

*For anti-Synapsin staining the following protocol was used*: Dissected brains were collected in glass wells containing 4% Formaldehyde in 0.3% PBT. For fixation brains were kept over night on a shaker in glass wells containing 4% Formaldehyde in 0.3% PBT (Triton X-100 in PBS) covered with parafilm. Antibody labeling was based on staining protocols used to label the adult *Drosophila* brain (Pereanu et al., 2010). Brains were incubated in primary antibodies diluted in 0.3% PBT containing 5% NGS (Normal Goat Serum, Vector, S1000) overnight at room temperature on a shaker covered with parafilm. Brains were washed 5 × 10 min, 4 × 60 min with 0.3% PBT at room temperature. For secondary antibodies the brains were incubated in secondary antibodies diluted in 0.3% PBT containing 5% NGS overnight at room temperature on a shaker covered with parafilm. Brains were washed 5 × 10 min, 4 × 60 min with 0.3% PBT at room temperature and moved to 80% glycerol or DAPI containing Vecatshield prior to imaging. As primary antibody anti-Synapsin was used at a final concentration of 1:10. As secondary antibodies we used the donkey anti mouse Alexa-555 (Molecular probes) 1:200.

*For anti-acetylated Tubulin staining the following protocol was used*: Dissected brains were collected in glass wells containing 4% Formaldehyde in 0.3% PBT. For fixation brains were kept for 30 min on a shaker in glass wells containing 4% Formaldehyde in 0.3% PBT (Triton X-100 in PBS) covered with parafilm. For washing steps and antibody incubation 3% PBT was used. After fixation the brains were rinsed twice with 3% PBT and then washed after 5 min, 10 min, 20 min, 30 min, 1 h, 2 h in 3% PBT. Brains were incubated in primary antibodies diluted in 3% PBT containing 5% NGS (Normal Goat Serum, Vector, S1000) overnight at 4°C on a shaker covered with parafilm. After primary antibody incubation the brains were rinsed twice with 3% PBT and then washed after 5 min, 10 min, 20 min, 30 min, 1 h, 2 h in 3% PBT. For secondary antibodies the brains were incubated in secondary antibodies diluted in 0.3% PBT containing 5% NGS at 4°C on a shaker covered with parafilm. After secondary antibody incubation the brains were rinsed twice with 3% PBT and then washed after 5 min, 10 min, 20 min, 30 min, 1 h, 2 h in 3% PBT. Brains were then transferred to 80% glycerol or DAPI containing Vecatshield prior to imaging. As primary antibody mouse anti-acetylated Tubulin (was used at a final concentration of 1:200. As secondary antibodies we used the donkey anti mouse Alexa-555 (Molecular probes) 1:200.

### Generation of 3D models

Immunostained adult brains were imaged and scanned as whole-mounts by confocal microscopy Leica SP5 (LASF software) 20× glycerol immersion objective using a “line average mode” with a picture resolution of 1024 × 1024 pixels. Complete series of optical sections were taken at 1–1.5 µm intervals. Digital images of confocal sections were imported into the freeware FIJI program. Complete series of optical sections were imported and processed using FIJI/ImageJ as previously described (Sprecher et al., 2006). 3D models were manually generated by using the TrakEM2 plugin. TrakEM2 is part of the FIJI software package, which can be accessed and downloaded here: http://pacific.mpi-cbg.de/wiki/index.php/Fiji.

## Results

### Compartmentalization of the *Cardiocondyla* brain

**Figure 1:**
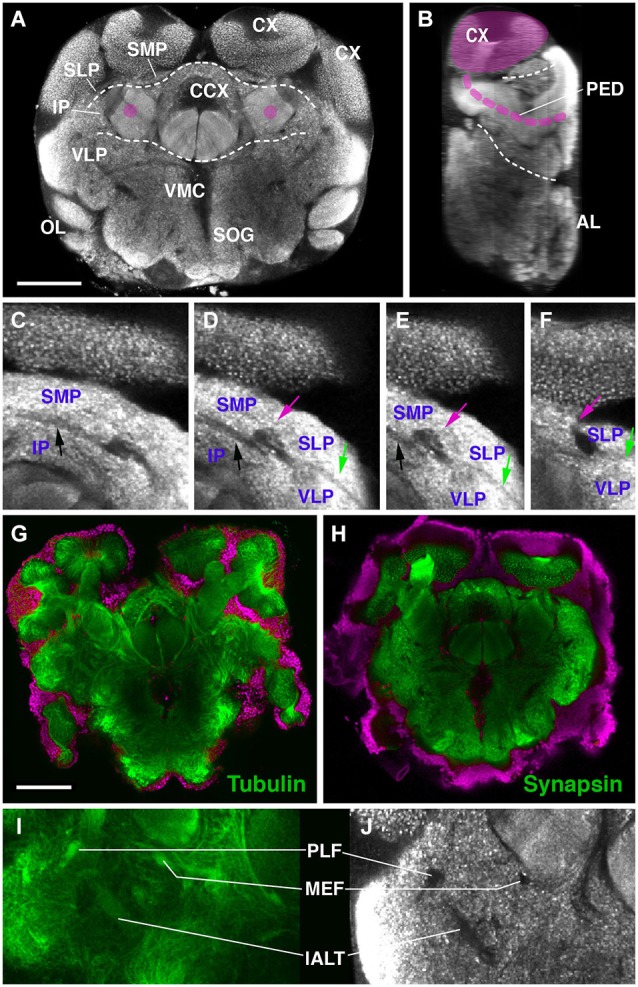
**Overview of the brain neuropil of *DrosophilaCardiocondyla obscurior* (C,D; labeled with anti-Synapsin). (A)** Cross section at level of central complex (CCX); **(B,D)** Sagittal section at level of peduncle (PED; anterior to the right). Section was derived by digital 90 deg tilt of the original confocal stack. Peduncle and calyx (CX) of mushroom body (MB) are shaded magenta. Hatched lines demarcate boundaries between dorsal layer, middle layer, and ventral layer of neuropile. **(C–F)** Consecutive cross sections of part of *Cardiocondyla* brain labeled with anti-Synapsin. Compartment boundaries stand out by low Synapsin signal. Boundaries between SMP and IP (black arrow), SMP and SLP (red arrow), SLP and VLP (green arrow). **(G–J)** Comparison of anti-Synapsin staining with anti-Acetylated-tubulin staining. **(G,I)** Cross section of the *Cardiocondyla* brain labeled with anti-Acetylated-tubulin (green). **(H,J)** Cross section at corresponding level, labeled with anti-Synapsin (green). Neuronal nuclei are labeled by DAPI **(G,H**; magenta). Synapsin-negative fiber bundles (PLF, MEF and l-ALT in **I,J**) are positively labeled by anti-Acetylated-tubulin **(I)**. For abbreviations see Table 1. Bar: 50 µm.

The definition of discrete compartments within the insect brain neuropil is based on long fiber systems and their associated glial sheaths. Neuropil compartments are rich in terminal neurites bearing synapses; long fiber tracts and glial processes, which are devoid of synapses, form boundaries around compartments (Pereanu et al., 2010). When using a marker for synapses, such as the anti-Synapsin antibody employed here, compartments stand out by a high signal level, whereas compartment boundaries are low in signal (Figures 1A–F). Anti-Synapsin has previously been applied successfully in other arthropods, including spiders and crustaceans (Fabian-Fine et al., 1999; Harzsch et al., 1999) to label the neuropil. In *Drosophila*, fiber bundles and glia can be positively labeled by a number of different antibodies and genetic markers. None of these markers cross reacted with epitopes of the *Cardiocondyla* brain (JB and SP, unpublished). However, we confirmed that Synapsin-negative spaces coincided with fiber systems by using an anti-acetylated Tubulin antibody. Axon bundles are typically rich in parallel running microtubules that are visualized with anti-acetylated Tubulin (Figures 1G–J). The nomenclature of compartments and fiber bundle systems employed in the following is based on previous studies done in several insect species, including Honeybee and *Drosophila* (Brandt et al., 2005; Menzel and Manz, 2005; Kirschner et al., 2006; Pereanu et al., 2010; Rybak et al., 2010; Rössler and Zube, 2011; Ito et al., 2014).

Neuropil compartments are grouped into three layers along the dorsal-ventral axis, operationally defined as the axis perpendicular to the MB peduncle, which is oriented from anterior (junction with lobes) to posterior (calyx) (Figures 1A,B). The dorsal layer is comprised of the medial and lateral superior protocerebrum (SMP, SLP), respectively. The middle layer includes the inferior protocerebrum (IP), MB, CCX and lateral accessory lobe (LAL). The ventral layer contains the AL, antenno-mechanosensory and motor center (AMMC), ventro-lateral cerebrum (VLP), postero-lateral cerebrum (PLP), ventro-medial cerebrum (VMC), and suboesophageal ganglion (SOG; Figures 3A–H).

**Figure 2:**
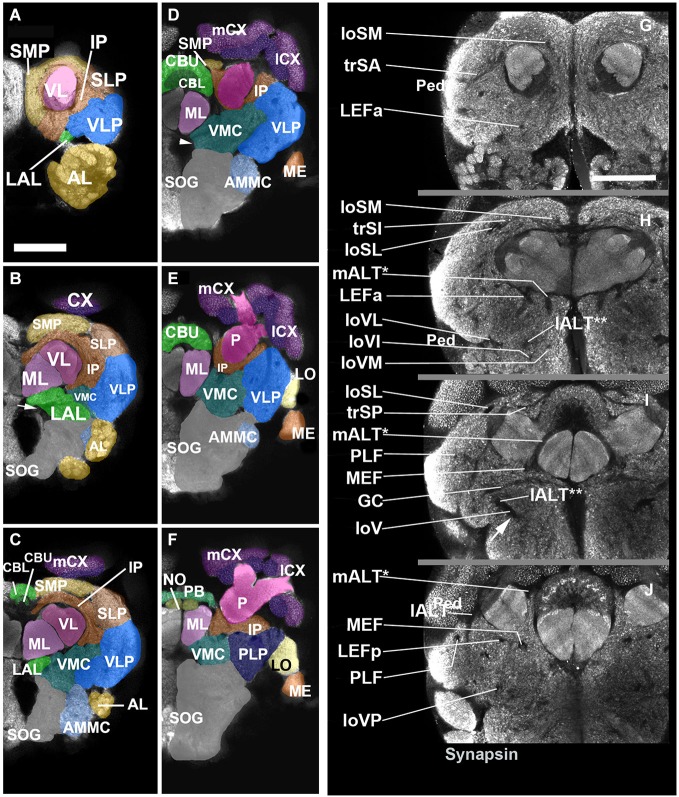
**Neuropil compartments of the *Cardiocondyla* brain and axon tracts associated with brain compartments. Panels (A–F)** show z-projections of 5–8 contiguous confocal sections of adult brain hemispheres labeled with anti-Synapsin (*Cardiocondyla*), demarcating neuropile compartments (shaded in different colors). Panels are ordered from anterior **(A)** to posterior **(F)**. **(A)** level of antennal lobe (AL); **(B)** level of MB medial lobe (ML); **(C)** level of the central body lower unit (CBL) ; **(D)** level of the central body upper unit (CBU) and great commissure (arrowhead); **(E)** level around posterior boundary of central body; **(F)** level of protocerebral bridge (PB). For abbreviations see Table 1. Panels **(G–J)** show individual confocal sections of *Cardiocondyla obscurior* stained with anti-Synapsin. Scalebar: 50 µm **(A–F)** and **(G–J)**, **(A–F)** and **(G–J)** are at same scale.

**Figure 3:**
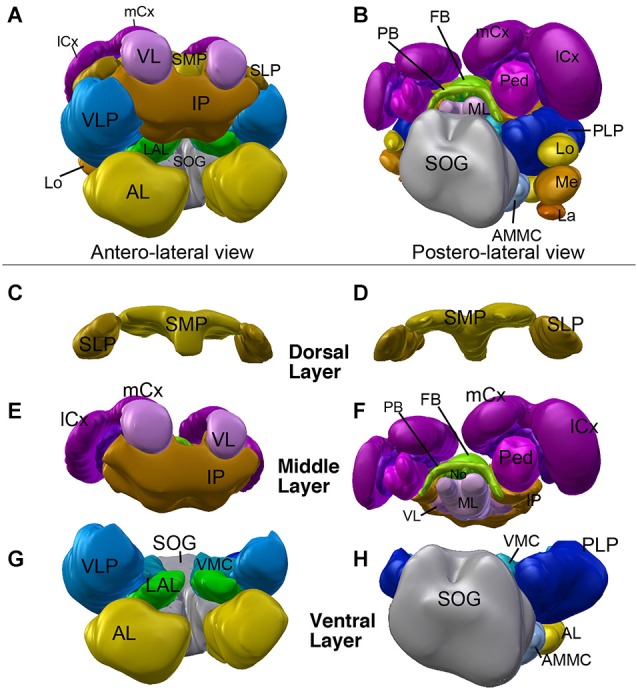
**3D digital model of the adult brain compartments of the ant *Cardiocondyla obscurior***. Colors of compartments correspond to those used in Figure 2. **(A,B)** Entire brain with the optic lobes; tilted anterior view **(A)** and posterior view **(B)**. Note the proximal-distal arrangement of the lobula complex, medulla and lamina. **(C–H)** Individual compartments of the three layers of the cerebrum; **(C,E,G)** antero-lateral view, **(D,F,H)** postero-lateral view. **(C,D)** Dorsal layer of neuropil, with the two compartments SMP and SLP. **(E,F)** Middle layers harboring the CCX (green) and mushroom bodies (magenta), and the inferior protocerebrum (IP; brown). Note massive MB calyces which are tilted dorsally and anteriorly, to cover much of the dorsal neuropil surface. **(G,H)** Ventral layer, composed of the lateral accessory lobe (LAL), ventromedial cerebrum (VMC), ventrolateral protocerebrum (VLP), posterolateral protocerebrum (PLP), AL, antennal mechanosensory and motor center (AMMC) and suboesophageal ganglion (SOG). For further abbreviations see Table 1.

### Compartment of the dorsal layer

#### Superior protocerebrum

The dorsal brain is composed of the superior medial and the superior lateral protocerebrum (SMP/SLP), and shows a dense anti-Synapsin immunoreactivity compared to the underlying IP (Figures 2A,B). At the neuropil surface a shallow furrow defines the boundary between the SMP and SLP (Figure 2C). Posteriorly, the arms of the peduncle, cupped by the two calyces, bound the superior protocerebrum; anteriorly, the SMP and SLP extend all the way to the anterior neuropil surface, enclosing the tip of the MB vertical lobe (Figure 2D).

### Compartments of the middle layer

#### The mushroom body (MB)

**Figure 4:**
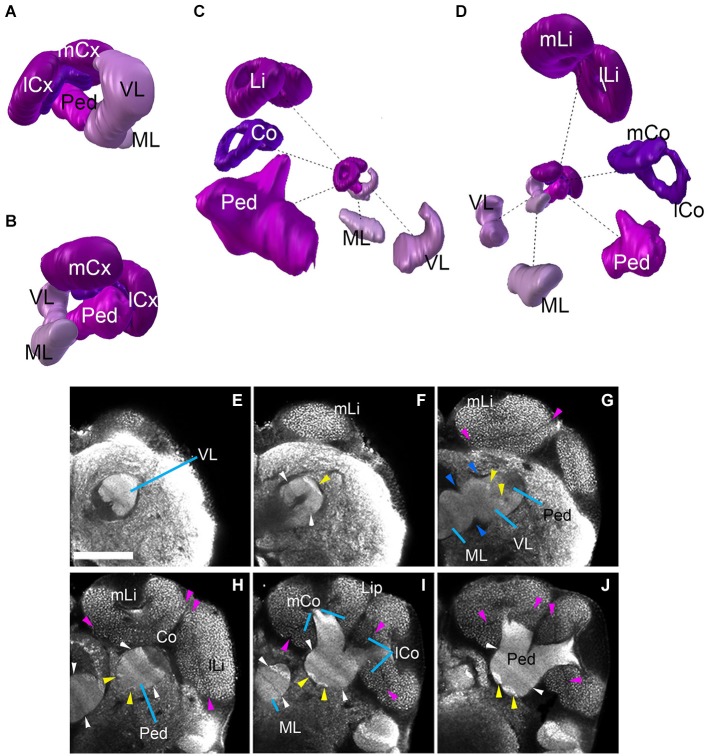
**Mushroom bodies neuropil complex of the ant** ***Cardiocondyla obscurior*. (A,B)** 3D digital model of the MB showing the relative position of each of its elements, **(C,D)** 3D model showing each of its elements isolated; note the bipartite organization of the calyx, the size of the different elements are not in scale. **(E–J)** show the MB elements on different Z-sections from anterior to posterior. Blue arrowheads show fringes between the lobes (medial and vertical) and Peduncle **(G)**. Pink arrowheads show the fringe between Lip and Collar. Yellow arrowheads indicate the bright hallmark continuous **(G,H)** between the VL and the Ped. White arrowhead **(F,H,I)** show the transition of brighter and less bright layers ML/VL and Peduncle. See discussion for more details. Scale bar: 50 µm. Abbreviations: MB: mushroom bodies, ML: medial lobe, VL: vertical lobe, Ped: peduncle, lCx: lateral calyx, mCx: medial calyx, Li: lip, Co: collar, mLi: medial lip, lLi: lateral lip, mCo: medial collar, lCo: lateral collar.

The mushroom body of *Cardiocondyla* shows the typical organization of Hymenopterans, with a vertical lobe (VL), a medial lobe (ML), and a peduncle (Ped) that splits into a medial and a lateral arm surrounded by a medial calyx and a lateral calyx (m-/l-Cx), respectively (Figures 2B–F, 3E,F, 4A–D). The lobes and the peduncle show an extremely fine-grained anti-Synapsin immunoreactivity suggesting densely packed, small diameter fibers, whereas the calyces appear granular due to the typical microglomerular organization (Stieb et al., 2010). Ped, VL and ML are organized into five conspicuous “slices” with alternating high and low levels of anti-Synapsin immunoreactivity (Figures 4E–I). The VL has a long, crescent-like shape, projecting dorsally and then posteriorly (Figure 4C). Similarly, the ML is bent posteriorly, reaching as far backward as the level of the calyces and protocerebral bridge (PB; Figure 2F).

The calyces form the distal-most part of the mushroom bodies and are the largest compartments of the hymenopteran brain, protruding far anteriorly over the dorsal surface of the brain (compare Figures 2A–F, 4A–D). In each brain hemisphere one distinguishes a medial and a lateral calyx, each exhibiting a typical doughnut-like shape (Figures 4C,D). Anti-Synapsin immunoreactivity is granular, reflecting the microglomerular organization of this structure (Figures 4E–J; Stieb et al., 2010). In the honey bee *Apis mellifera*, the calyces were subdivided into three distinct domains, termed the basal ring (Br), collar (Co) and lip (Li). It has been reported that this arrangement is maintained in ants, but the presence as well as the position of single sub-compartments depends on the species (Gronenberg, 2001). The calyces of *Cardiocondyla* show two clear domains with different macroglomerular size and organization, a thick upper domain, the lip, and a lower smaller domain, the Co; we do not find an anti-Synapsin-based criterion that would allow us to distinguish a Br (Figures 4A–D,H,I).

#### The central complex (CCX)

**Figure 5:**
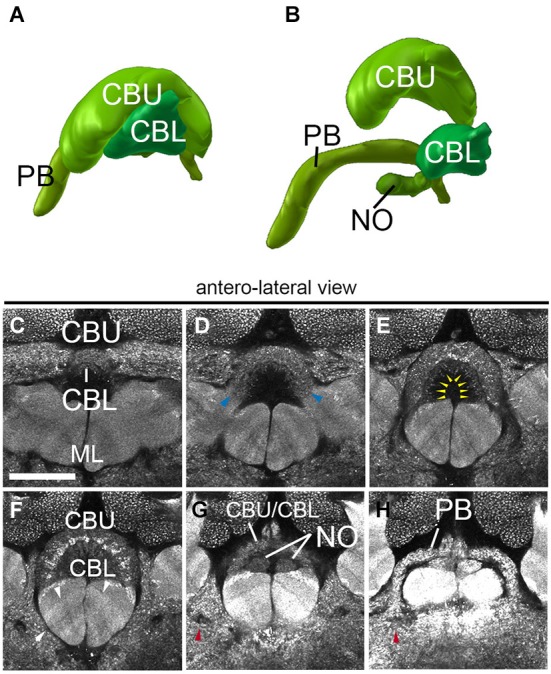
**Central complex neuropils of the ant** ***Cardiocondyla obscurior*. (A,B)** 3D digital model of the different neuropil compartments of the CCX. **(C–H)** Confocal sections of the CCX, **(C–F)** Central body: central body upper unit (Fan-shaped body) and central body lower unit (ellipsoid body). **(G)** transition between the Fan-shaped body and the Protocerebral bridge. **(H)** Protocerebral bridge. Blue arrowheads in **(D)** show the fringe between the CBU and CBL. Yellow arrowheads in E tentatively label the subregions of the CBU/CBL. Red arrowhead in **(G)** and **(H)** show the endpoint of the MEF under the PB. Arrows in F show the layering of the underlying medial lobe. Scale bar: 50 µm.

The central complex, a prominent neuropil assembly shared by all insects, is composed of the central body (CB) with an upper (CBU) and a lower unit (CBL; corresponding to the fan-shaped body and ellipsoid body in Dipterans, respectively), the associated PB and the noduli (NO; Strausfeld, 2012). The central body has the shape of a short hemicylinder located at the dorsal midline of the brain (Figures 2C–F, 5A,G,H). Anti-Synapsin staining clearly reveals the modular structure and layering of the central body. Based on anti-Synapsin signal intensity and texture one can distinguish an upper unit (=fan-shaped body), and a lower unit (=ellipsoid body; Figures 5A–F). At a central level (Figure 5F) where it reaches its greatest diameter, the upper unit is further subdivided into a wider dorsal layer of moderate synaptic density, and a thin middle layer with high density. Along the (curved) medio-lateral axis, both the upper and the lower unit of the central body are divided into eight modules, four on each side (Figures 5D–F), matching a similar modular structure described for Dipterans (Young and Armstrong, 2009) and other species (Boyan and Williams, 1997; Williams et al., 2005). The NO are attached to the posterior surface of the CBL (Figures 5B,G,H).

#### Inferior protocerebrum (IP)

The inferior cerebrum surrounds the peduncle (medial and lateral inferior protocerebrum) and lobes of the MB (anterior inferior protocerebrum). At an anterior level, the IP is flanked laterally by the VLP, ventrally by the LAL and dorsally by the SMP and SLP (Figures 2A–F). Posteriorly the IP is represented by thin plates of neuropil flanking the peduncle medially and laterally, respectively (Figure 2E).

### Compartments of the ventral layer

The topology of the ventral brain can be illustrated by a cross section through the center of the brain, as shown in Figures 2D,F. Two thick commissural tracts, the great commissure (GC) and the dorsal commissure of the suboesophageal ganglion (DC-SOG), as well as a group of longitudinal fiber bundle, forming the longitudinal ventral fascicle (loV), enclose the ventromedial cerebrum (VMC). It is flanked ventrally by the SOG, laterally by the ventro-lateral protocerebrum (VLP), and anteriorly by the LAL. Two sensory compartments, the AL and AMMC tip the anterior surface of the ventral brain.

#### Antennal lobe

The antennal lobe (AL) functions as an integration center for the olfactory information coming from the antennae, and it is connected by projection neurons to the calyx of the MB (Galizia et al., 1998; Galizia and Rossler, 2010). As in other insects surveyed in the literature, the AL is formed by several hundreds of glomeruli of different size and shape, arranged around an inner core of fibers and neuropil (Figures 2A,B). Output fibers gather towards the center of the AL and project dorso-posteriorly, forming the conspicuous medial AL tract (see below).

#### Antenno-mechanosensory and motor center (AMMC)

The AMMC, a compartment receiving input from the mechanosensory afferents of the head, including the Johnston’s organ (the “ear”) of the antenna, is situated posterior of the AL. It does not show a glomerular architecture. Deep surface furrows delineate the AMMC from the neighboring SOG (medially) and ventro-lateral protocerebrum/optic lobe (laterally; Figures 2C,D). Posteriorly, the AMMC merges smoothly with the SOG.

#### Lateral accessory lobe (LAL)

The LAL, formerly called “ventral body” in some of the classical papers, is closely associated with the CCX. We identified a compartment located in between the AL (antero-ventrally), anterior inferior protocerebrum (dorsally) and VLP (laterally) as the *Cardiocondyla* LAL (Figures 2B,C). A thick, straight commissure crossing right anterior to the upward sweeping AL tract, the commissure of the LAL (LALC), forms a characteristic landmark for the LAL (arrows in Figures 2B,D, respectively). While clear furrows at the neuropil surface separate the LAL from its neighboring compartments the posterior boundaries of the LAL are less well defined, thus the LAL in *Cardiocondyla* may at this stage not better depicted.

#### Ventromedial cerebrum (VMC)

The VMC is the neuropil compartment surrounding the GC and can be divided into a pre-commissural domain (anterior to the GC; VMCpr), commissural domain (VMCc; ventral of GC), and post-commissural domain (VMCpo). At its dorsal surface the VMC is bounded by the MB and IP; laterally, fiber bundles devoid of anti-Synapsin immunoreactivity demarcate the border between VMC and VLP (Figures 2B–F). The VMC flanks the foramen of the esophagus. This opening is wide at anterior levels of the brain, but becomes very narrow towards posteriorly (Figure 2F).

#### Ventrolateral protocerebrum

The ventro-lateral protocerebrum represents the domain of the central brain that receives visual input from the optic lobes. In Dipterans, terminal arbors of optic lobe projection neurons form conspicuous, glomerulus-like condensations, called optic foci (Mu et al., 2012). Optic foci could not be detected in anti-Synapsin labeled material of *Cardiocondyla*, At central levels (right posterior to the great commissure, Figures 2E,F), the VLP bulges laterally and merges with the lobula complex of the optic lobe (Figure 2E).

#### Suboesophageal ganglion and adjoining regions

The SOG is formed by four fused neuromeres, belonging (from anterior to posterior) to the intercalary segment (this neuromere, when morphologically distinct, is called the tritocerebrum), the mandibular segment, maxillary segment, and labial segment. The SOG receives sensory input from the mouth cavity (pharyngeal nerve) and the appendages grouped around the mouth, and involved in feeding (Homberg and Hildebrand, 1989; Eichmüller et al., 1991; Rajashekhar and Singh, 1994; Sun et al., 2003; Ignell and Hansson, 2005). In *Cardiocondyla*, the SOG takes up a relatively large volume, but currently no major landmarks were identified to further delimit its organization (Figures 2B–F).

### Optic Lobe Neuropils

In many insects, including Dipterans and many Hymenopterans, the optic ganglia form a dominating structure of the adult brain. However, the eyes of *Cardiocondyla* are comparably small, which is reflected in a similarly reduced size of the optic ganglia. They are located ventro-anteriorly of the PLP. From proximal to distal one can distinguish the lobula complex (LO), Medulla (ME) and lamina (LA; Figure 3B). The LO remains as a globular compartment tightly associated to the VLPp and shows one layer of dense anti-Synapsin immunoreactivity. The ME is wider along the anterior-posterior axis compared to the LO. Its middle domain is dorsally attached to the LO. By means of anti-Synapsin immunoreactivity we can identify a clearly layered structure of the medulla from proximal to distal. The ME has an ellipsoidal shape and is extended along the dorso-medial axis. The distalmost part of the OL is the LA having a rather horizontal orientation and spanning the ME in the posterior region (not shown).

### Major fiber systems of the central brain

A large number of long axons that interconnect different regions of the brain form tight bundles (fascicles). These neuropil fascicles, aside from compartments, represent an important set of landmarks that can be referred to when comparing brains of different insects. In brain preparations labeled with markers for synapses, like anti-Synapsin in this study, axon bundles appear as signal-negative channels. We confirmed that these channels are indeed fiber bundles by using an anti-acetylated Tubulin antibody, which labels the dense arrays of microtubules within axons we confirmed that Synapsin signal-negative areas are indeed fiber bundles (see above; Figures 1G,I). Since anti-Synapsin stained preparations are richer in landmarks, also depicting neuropil domains that are separated by cell bodies and glia sheets, we used this staining to define the pattern of fascicles.

Most fascicles are grouped along the boundary separating the IP from the compartments of the superior protocerebrum and the ventral cerebrum. We can distinguish three main system of fibers that run along compartment boundaries: a set of dorsal (“superior”) superior fascicles, extending along the SP/IP boundary; a system of longitudinal fascicles between the IP and the ventral cerebrum (“equatorial fascicles”); a ventral system of longitudinal and transverse fascicles (among them the great commissure and commissure of the LAL) associated with the ventral brain compartments (Figures 2G–J).

#### The superior fascicles

**Figure 6:**
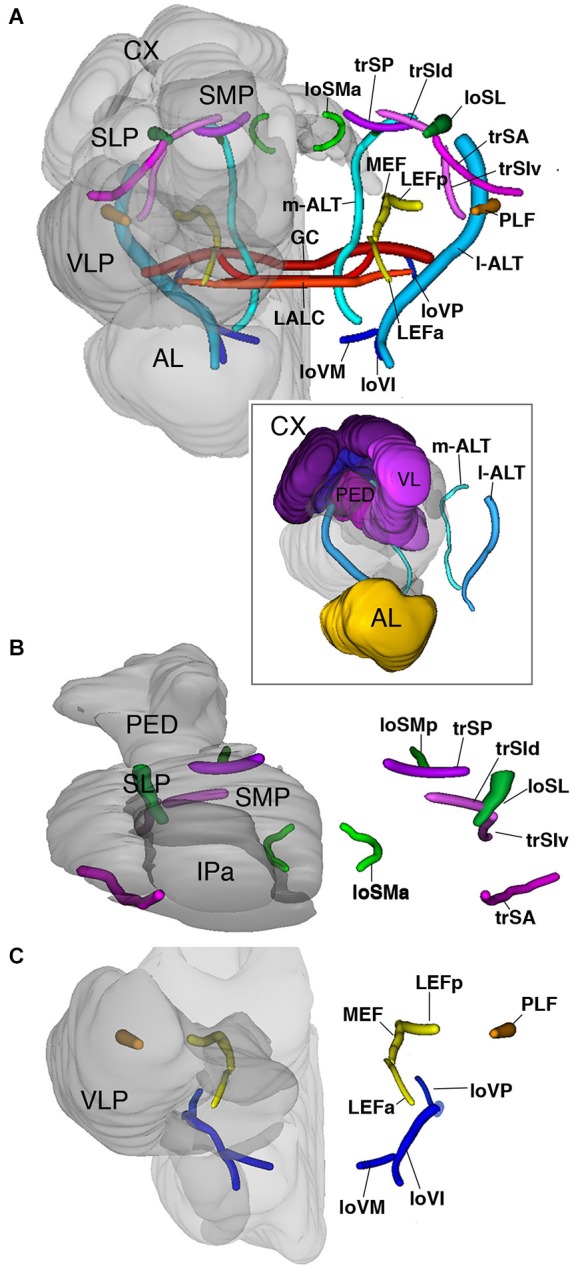
**3D digital rendering of the long axon fascicles**. Longitudinal fascicles of dorsal neuropil: green; transverse fascicles of dorsal neuropil: magenta; longitudinal fascicles of middle neuropil: yellow (LEF, MEF), orange (PLF); longitudinal fascicles of ventral neuropil: blue; AL tracts: cyan; ventral commissures: red. **(A)** Anterior view of all fascicles. For left hemisphere, neuropile compartments (gray, semi-transparent) are superimposed upon fascicles. Boxed inset: anterior view of AL tracts in relationship to MB. **(B)** Dorsal view of fascicles of dorsal neuropil. **(C)** Anterior view of fascicles of middle and ventral neuropil layers. For further abbreviations see Table 1.

The superior system is composed of three transverse superior fascicles, called transverse superior anterior (trSA), transverse superior intermediate (trSI) and transversal superior posterior (trSP) fascicle (Figures 2G–I, 6A,B). The longitudinal systems include a lateral and medial longitudinal superior fascicle (loSL, loSM; Figures 2G–I, 6A,B). The anterior portion of the loSM (loSMa) enters the antero-dorsal surface of the neuropil medially to the tip of the vertical lobes. It extends posteriorly along the SMP-IP boundary, remaining medial to the VL (Figures 2G,H, 6B). The posterior component of the loSM (loSMp) enters the postero-dorsal neuropil in the small triangular “window” flanked by the medial calyx (dorsally), peduncle (laterally), and fan-shaped body (medially (Figure 6B). The loSMp projects anteriorly over a short distance before merging with the posterior transverse superior fascicle (trSP; Figure 6B).

The longitudinal superior lateral fascicle (loSL; Figures 2H,I, 6A,B) is thicker and longer than its medial counterpart. It enters the posterior neuropil dorsally to the peduncle. The loSL follows the boundary between SLP and SMP (Figure 2I) and continues anteriorly before terminating in the anterior IP (Figure 2I).

Among the superior transverse fascicles, the trSA enters the lateral neuropil surface anteriorly (vertical lobe, antennal lobe) and extends along a crescent-shaped trajectory antero-dorso-medially, to reach the posterior surface of the vertical lobe (Figures 2G, 6B). The trSA defines the boundary between SLP (above) and VLP/IP (below). The intermediate transverse tract, trSI, enters into the neuropil posterior to the trSA. It continues straight dorso-medially, passing the loSL fascicle, and forming the boundary between superior and IP (Figures 2H, 6B). The trSP is found posteriorly, at the level of the distal peduncle and fan-shaped body, forming a crescent-shaped connection between the loSL and the dorso-medial neuropil surface (Figures 2I, 6B).

#### The equatorial fascicles

A set of longitudinal fiber system extends along the ventral boundary of the IP. The medial equatorial fascicle (MEF) is a large fiber system that enters the posterior neuropil surface right at the lateral tip of the PB (Figures 2I,J, 6A,C). It projects straight anteriorly, parallel to the ventro-medial surface of the peduncle, the inferior protocerebrum (IP; above) and ventromedial cerebrum (VMC; below). At the level of the GC the MEF approaches the peduncle, close to the position where the ML buds off. This position is marked by a conspicuous indentation in the surface of the peduncle/medial lobe (“peduncle-ML-groove”; Figure 2J). Continuing anteriorly into the LAL, the MEF turns medially and merges with the commissure of the LAL.

The lateral equatorial fascicle (LEF) represents a thinner fiber bundle located lateral of the MEF. The posterior component of the LEF (LEFp) enters the posterior brain surface adjacent to the MEF (Figures 2J, 6A,C). The LEF extends anteriorly and converges onto the ventral surface of the peduncle. One can recognize an anterior component of the LEF (LEFa), which enters the neuropil in the depth of the furrow between the LAL and VLP (Figures 2G, 6A,C), projects posteriorly to meet the posterior LEF/MEF at the peduncle-ML groove.

Lateral to the LEF is a complex system of longitudinal bundles, which (in part) may correspond to the system called “postero-lateral fascicle” (PLF). Two thick bundles, one near the lateral neuropil surface, the other more medial, close to the LEF, enter the posterior lateral protocerebrum and follow a straight anterior trajectory towards the anterior reaches of the IP and laterally adjacent VLP (Figures 2I,J, 6A,C), where they end. A third bundle, even larger in diameter, begins further dorsally, but sweeps antero-ventrally, to connect to the longitudinal ventral system.

#### The ventral fascicles

The longitudinal ventral system of fiber bundles enters the brain ventral to the AL and the AMMC. It consists of a medial (loVM), intermediate (loVI) and lateral (loVL) component. We recognize a relatively thin medial bundle (loVM) which enters posterior to the AL at the boundary between the anterior tip of the SOG and the laterally adjacent AMMC and VMC (Figures 2H, 6A,C). From here the loVM projects postero-laterally, to end in the ventral zone (arrow in Figure 2I). Further laterally, two additional bundles enter the AMMC and converge upon the ventral zone. The thicker, dorsal one of these projects through the dorsal AMMC; the ventral one enters the AMMC at its center (Figure 2H).

#### The antennal lobe tracts

These fiber systems, which are also called antenno-glomerular, antenno-cerebral, or antenno-protocerebral tracts in the literature, are part of the olfactory pathway and connect the AL to the MB complex and adjoining superior protocerebral domains. We employ the term “antennal lobe tract” as recently justified in Ito et al. (2014). Hymenopterans, like Dipterans, have a dual olfactory pathway with a medial and a lateral antennal lobe tract (resp. m-ALT and l-ALT) (Kirschner et al., 2006). They also have a medio-lateral ALT (ml-ALT) that project directly into the lateral horn (Zube and Rössler, 2008; Rössler and Zube, 2011). In anti-Synapsin labeled preparations of the *Cardiocondyla* brain we clearly identified the m-ALT and l-ALT, while the ml-ALT was less obvious. The m-ALT starts from the AL and extends posteriorly through the LAL. After passing the LALC commissure the m-ALT turns upward and enters the groove between the ML and VL, from where it projects dorsally towards the calyces. It exits the dorsal surface of the neuropil between the ML and the IP and terminates ventral to the Co (Figures 2H, 6A). The l-APT appears thick and starts from the AL to its exit point of the neuropils after crossing the PLF under the Co (Figure 6A).

#### The commissural systems

Commissures in the fly brain are grouped around the dorsal surface and ventral surface of the CCX and MLs of the MB; in addition, several commissures cross at the level of the ventral cerebrum (Strausfeld, 1976; paper by Boyan on grasshopper commissures). The commissural tracts surrounding the CCX can be individualized only if labeled positively with axonal markers. In preparations labeled with a synapse marker like anti-Synapsin, both commissural tracts and the massive glial sheaths surrounding the CCX coalesce into signal-negative layers (Figures 2H,I), and it is not possible to pin-point individual commissural tracts.

Three commissures connect ventral brain compartments, namely (from anterior to posterior) the LAL commissure (LALC; interconnecting LAL hemispheres), the great commissure (GC; interconnecting ventral compartments), and the posterior PLP commissure (posterior-lateral protocerebrum). Both LAL commissure (Figure 2, arrows) and GC (Figure 2I) could be identified at their characteristic positions in *Cardiocondyla*.

## Discussion

Neuropil compartments are signal-processing centers, which receive inputs from sensory neurons or interneurons of other brain domains. Compartments together with the systems of long axon tracts by which they are interconnected represent elements of the “macrocircuitry” of the insect brain. A compartment map provides the framework to which the connectivity of individual neurons can be referred. With this idea in mind, more or less detailed compartment maps have been generated for numerous species, including the fruit fly (Rein et al., 2002; Ito et al., 2014), the hawkmoth (El Jundi et al., 2009b), the desert locust (Kurylas et al., 2008; El Jundi et al., 2009a), the red flour beetle (Dreyer et al., 2010) and the monarch butterfly (Heinze and Reppert, 2012; Heinze et al., 2013). In this paper we provide an anatomical map of the brain of the worker cast of the social ant, *Cardiocondyla obscurior*.

### Structured neuropil compartments of the central brain: mushroom body, central complex, and antennal lobe

While MBs may display discrete anatomical differences between different insect species, homologies are detectable at many levels (Strausfeld et al., 2009). The MB of *Drosophila* and other Dipterans is relatively small and is thought to primarily process olfactory information. In social Hymenoptera, including *Cardiocondyla*, the MB is strongly enlarged compared to flies, and receives more widespread input from the optic lobe. The two modalities, olfaction and vision, are segregated in the calyx. The calyx of the honeybee has three main regions: the distally located lip (olfactory input region), the Co, located beneath the lip (visual input region), and the Br (input from both modalities). While the *Cardiocondyla* worker caste has comparably small eyes the eyes of winged males and queens are large. Comparison of the relative size of distinct calyx regions between large-eyed and small-eyed castes may give further insight into how these domains are differentially employed in distinct morphs. Axonal endings of Kenyon cells associated with the three calycal subdomains form three separate layers in the vertical lobe; a fourth layer is represented by the γ-layer (Fahrbach, 2006). In ants, the transition between the lip and Br is smoother than in honeybee. In most members of the subfamily Myrmicinae, lip and Br appear fused; only in *Aphaenogaster boulderensis*, a distinct part of the calyx domain has been tentatively identified as Br (Gronenberg, 2001). In *Cardiocondyla*, the calyx lip and Co can be clearly distinguished by macroglomerular size and organization, but we could not unambiguously identify a separate Br.

In terms of relative size and internal composition, the compartments of the CCX of *Cardiocondyla* corresponds closely to that described for other insects. Along the dorso-ventral axis distinct layers can be recognized on the basis of synapse density. The number of layers appears to vary widely among insects; the *Drosophila* fan-shaped body, for example, has seven or eight layers (Young and Armstrong, 2009; Ito et al., 2013), while only two layers are visible in the upper division of the central body in *Cardiocondyla* (this study). In contrast, the subdivision of the central body along the transverse axis into eight regular columns is conserved between *Drosophila*, *Cardiocondyla*, and other insect species (Williams et al., 2005; Homberg, 2008).

The olfactory systems in honeybees and the ants *Camponotus japonicus* and *Atta vollenweideri* show glomeruli further organized into clusters that correspond to the subdivisions of the antennal nerve into distinct tracts. In the honeybee the antennal nerve is split into four main tracts (T1-4) (Abel et al., 2001), and the T3 tract can be further divided into three bundles (T3a-c), thus subdividing the AL into a total of six clusters (Kirschner et al., 2006). In the ant *Camponotus japonicus* the antennal nerve is divided into seven tracts (T1-7), thereby defining seven glomerular clusters (Zube et al., 2008); in workers of the ant *Atta vollenweideri* only six clusters (T1-6) have been identified. In *Cardiocondyla* we recognize five glomerular clusters. To further characterize the *Cardiocondyla* AL in the future, nerve backfilling studies might give deeper insight. The size of antenna in the four *Cardiocondyla* castes is strikingly different. Antennae of winged males are substantially larger than those of all other castes, likely due to their function in detecting flying queens and proper courtship behavior. This difference in antenna size also correlates with anatomical differences in the AL of winged males (Bressan et al., personal observation).

### Unstructured neuropils of the *Cardiocondyla* brain

Neuropil compartments that do not stand out by possessing repetitive or symmetric elements are traditional called “unstructured”. Even though they account for most of the neuropil volume of the central insect brain, these compartments have not received much attention; their boundaries were not defined, no consistent nomenclature has been proposed, and they are generally not included in the 3D digital models that were generated for insect “model species” like honeybee (Brandt et al., 2005; Rybak et al., 2010), desert locust (El Jundi et al., 2009a), red flour beetle (Dreyer et al., 2010). The atlas of the *Musca* brain (Strausfeld, 1976) places topologically based annotations (e.g., superior lateral protocerebrum, inferior protocerebrum) over these unstructured neuropil domains without attempting to define clear compartment boundaries. By utilizing markers that visualize synapses, glia and long fiber tracts one can distinguish between neuropil domains rich in terminal neuronal fibers and synapses (compartment centers) from other domains that contain long axon tracts associated with glial processes (compartment boundaries), a tentative map of unstructured neuropil compartments was generated for the *Drosophila* brain (Pereanu et al., 2010; Ito et al., 2014). Using similar markers, one should be able to reconstruct a more comprehensive compartment map for other insects as well. Our paper is a first attempt to delineate boundaries within the unstructured neuropil in the ant *Cardiocondyla*.

The relative size of the superior medial and lateral protocerebrum are significantly smaller in *Cardiocondyla* as for instance in *Drosophila*. In the *Drosophila* brain, these neuropil domains form conspicuous bulges at the dorsal brain surface (Strausfeld, 1976); in *Cardiocondyla*, they appear as relatively flat structures separated by a shallow groove. Similarly, other prominent bulges that deform the dorsal surface of the protocerebrum in other species (e.g., the lateral horn and the optic tubercles) do not exist in *Cardiocondyla*. This does not imply that specific neuropil compartments are missing; it merely suggests that the number of neurons contributing to that compartment may be reduced in comparison to other species, such as *Drosophila*. For example in the lateral horn compartment, which is formed by afferent lineages of antennal projection neurons, and local interneurons. In the *Drosophila* larva, a single lineage of about 20 neurons provide antennal lobe-derived input to the lateral horn (Python and Stocker, 2002; Das et al., 2013). In the adult, this number increases to three lineages totaling several hundred neurons; in addition, several hundred local interneurons restricted to the lateral horn are added during metamorphosis (Ito et al., 2013; Wong et al., 2013; Yu et al., 2013). In the larval brain, the protocerebrum has a smooth, convex surface; the “lateral horn circuitry” can only be revealed by specifically labeling AL afferents, or interneurons. The lateral horn bulge appears during the first two days of metamorphosis when secondary lineages mature. Correspondingly, ablation of secondary lineages results in the lack of the lateral horn bulge (Lovick et al., 2013).

It is currently not clear what the significant size differences in individual brain compartments mean, mostly because the function of these compartments is unknown. As indicated above, the size of dedicated sensory compartments, such as the AL or optic lobe, clearly correlates with the amount of sensory input, which in turn is reflected in the resolution of olfaction, or vision. Likewise, the size of the MB is commonly correlated with the ability of acquisition and storage of learned information, which is particularly high in social insects (Strausfeld, 2002; Ehmer and Gronenberg, 2004). However, what does the relative increase or decrease in size of the superior and IP signify? These compartments do not receive direct sensory input. We anticipate that the mapping of brain lineages and their fiber tracts, which has gathered momentum in recent years in *Drosophila*, will help in the effort to address the questions of functional brain anatomy. For example, it has become clear that a large number of secondary lineages (which contain roughly 90% of the neurons) form connections within and in between the ventrolateral protocerebrum (which is targeted by the optic lobe) and the superior protocerebrum, including the lateral horn (Ito et al., 2013; Yu et al., 2013). This macro-anatomical finding suggests that the lateral horn, which of course also receives olfactory input, and superior protocerebrum play a role in higher order processing of visual information. Given the relatively small size of the *Cardiocondyla* optic lobe one may speculate that the reduction of the superior protocerebrum/lateral horn may reflect a paucity in visual input and visually guided behaviors.

### Outlook

The generation of a complete 3D atlas of the neuropil compartments and fiber tracks in *Cardiocondyla* worker brain represents an entry point into a detailed characterization of the brain in this ant. The peculiar female and male polyphenism of *Cardiocondyla* and the corresponding behavioral repertoire is likely represented in the neuroarchitecture of specific brain domains. For instance, the ability of winged morphs to fly is accompanied with an increased ommatidia number in the eyes. Both winged males and queens also have ocelli at the top of their head, known to function for navigation in a three dimensional space. Thus, optic neuropils and the corresponding higher brain centers are larger in size in queens and winged males (Bressan and Sprecher, unpublished observation). The worker brains represent a simpler but more specialized state compared to the brains of queens and winged males. Future investigations of the central brain compartments in all four *Cardiocondyla obscurior* morphs will provide insight into the plasticity of the brain.

## Conflict of interest statement

The authors declare that the research was conducted in the absence of any commercial or financial relationships that could be construed as a potential conflict of interest.

## Abbreviations

**Neuropil compartments**: MB: Mushroom body
VL: Ventral lobe
ML: Medial lobe
Cx: Calyx
Br: Basal ring
Co: Collar
Lip: Lip
m-Cx: Medial calyx
l-Cx: Lateral calyx
Ped: Peduncle
CCX: Central complex
CB: Central body
CBU: Central body upper unit
CBL: Central body lower unit
PB: Protocerebral bridge
No: Noduli
IP: Inferior protocerebrum
mIP: Medial inferior protocerebrum
lIP: Lateral inferior protocerebrum
aIP: Anterior inferior protocerebrum
SP: Superior protocertebrum
SMP: Superior medial protorcerebrum
SLP: Superior lateral protocerebrum
AL: Antennal lobe
aAL: Anterior domain
vAL: Ventral domain
dAL: Dorsal domain
mAL: Medial domain
lAL: Lateral domain
AMMC: Antenno-mechanosensory and motor center
LAL: Lateral accessory lobe
VMC: Ventromedial cerebrum
VMCpr: Pre-commissural domain
VMCpo: Post-commissural domain
VLC: Ventrolateral cerebrum
SOG: Suboesophageal ganglion
OL: Optic lobe
La: Lamina
Me: Medulla
Lo: Lobula complex
**Axon tracts and fascicles**: trSA: Transverse superior anterior fascicle
trSI: Transverse superior intermediate fascicle
trSP: Transversal superior posterior fascicle
loSL: Lateral longitudinal superior fascicle
loSMa: Anterior medial longitudinal superior fascicle
loSMp: Posterior medial longitudinal superior fascicle
MEF: Medial equatorial fascicle
LEF: Lateral equatorial fascicle
loVM: Medial longitudinal ventral fascicle
loVI: Intermediate longitudinal ventral fascicle
loVL: Lateral longitudinal ventral fascicle
m-ALT: Medial antennal lobe tract
l-ALT: Lateral antennal lobe tract
GC: Great commissure
SEC: Supra-ellipsoid body commissure
SuEC: Sub-ellipsoid body commissure
SAC: Superior arch commissure
dPLPC: Dorsal PLP commissure
ALC: Antennal lobe commissure.
